# Usability of a device for lip strength and mobility rehabilitation associated with digital games: a pilot study

**DOI:** 10.1590/2317-1782/e20240271en

**Published:** 2025-10-20

**Authors:** Joyce Marques Barroso, Clarice Magnani Figueiredo, Eduardo Pena Castro Fantini, Marcos Antônio Abdalla, Andréa Rodrigues Motta, Estevam Barbosa Las Casas, Renata Maria Moreira Moraes Furlan

**Affiliations:** 1 Programa de Pós-graduação em Ciências Fonoaudiológicas, Universidade Federal de Minas Gerais – UFMG - Belo Horizonte (MG), Brasil.; 2 Programa de Pós-graduação em Engenharia de Estruturas, Universidade Federal de Minas Gerais – UFMG - Belo Horizonte (MG), Brasil.; 3 Wildlife Studios - São Paulo (SP), Brasil.; 4 Serviço Nacional de Aprendizagem Industrial - SENAI SAT - São João Del Rei (MG), Brasil.; 5 Departamento de Fonoaudiologia, Universidade Federal de Minas Gerais – UFMG - Belo Horizonte (MG), Brasil.; 6 Departamento de Engenharia de Estruturas, Universidade Federal de Minas Gerais – UFMG - Belo Horizonte (MG), Brasil.

**Keywords:** Muscle Strength, Video Games, Rehabilitation, Lips, Myofunctional Therapy

## Abstract

**Purpose:**

To evaluate the usability of a device for lip strength and mobility rehabilitation, combining exercises with digital games, and to examine the influence of age, muscle tension, and the number of attempts on the performance of adults and children.

**Methods:**

This observational, cross-sectional study included 11 adults and nine children. Participants used a device consisting of an anatomical-functional prototype for the rehabilitation of the orbicularis oris muscle, which works as a controller for digital games and is activated by the counter-resistance movement performed by the lips. Participants played a game with the device and received a score proportional to their performance. After using the game, adult participants completed the System Usability Scale (SUS) to assess the device's usability. Participants' scores were compared based on age, lip muscle tension, and the number of attempts.

**Results:**

Usability was considered good, with a mean score of 91.1 and a standard deviation of 11 points. There was no difference in scores across different attempts or between the scores of adults under and over 22 years old or children with normal and reduced lip tension. A statistically significant difference was found between adults’ and children’s scores in the first attempt and the mean of the attempts.

**Conclusion:**

The device demonstrated good usability, with age (adults vs. children) influencing participant scores.

## INTRODUCTION

Technological advances in health sciences, and specifically in speech-language-hearing therapy, improve assessment and therapeutic strategies used during interventions to rehabilitate the stomatognathic system muscles. Game therapy is one such strategy that can be used to enhance patient interaction^(1,2)^.

Game therapy uses "serious games," a category developed for both the entertainment of its users and intervention and rehabilitation in the case of impairments^(3)^. The objective of "serious games" is to promote more playful and attractive activities focused simultaneously on learning, training, and developing specific skills^(2)^. Some of its fundamental elements are stimulating cognitive functions, motivating, and constructing new knowledge^(4)^.

The use of digital games in conjunction with myotherapy can improve patient adherence^(5)^ and promote changes not only at the muscular level but also in the motor cortex^(6)^. Furthermore, this type of innovation can improve the quality of care by entertaining and amusing users, especially children^(7)^, which is why they are increasingly explored in motor training programs^(8,9)^.

Various studies in oral-motor therapy practice have developed and presented computer games associated with tongue exercises^(10-13)^. However, no studies were found associating computer games with lip rehabilitation. The lips are responsible for important body functions, and changes in their muscle tone/tension hinder the performance of chewing, swallowing, and speech^(14)^. Lip hypotonia is common in clinical conditions such as mouth breathing^(4)^, facial paralysis^(15)^, dental malocclusion^(16)^, neurodegenerative diseases^(17,18)^, and dysphagia^(19)^. These conditions require muscle training to regain strength^(20)^.

A device for rehabilitating lip strength and mobility^(21)^ has been recently developed in Brazil, combining myotherapy exercises with digital games. The device consists of an instrument that serves as a console for digital games, with which the user can execute commands with their lips according to the game's demands, previously adjusted by the therapist^(21)^.

However, the device’s usability has not been evaluated, which, according to the International Organization for Standardization (ISO)^(22)^, is related to the efficiency, effectiveness, and satisfaction of how an individual interacts with the product to achieve specific goals. The main usability evaluation methods use data from users or experts in the field^(23)^. Usability evaluation verifies whether the system meets the user's real needs, identifies flaws, and enables corrections for a better user experience.

Hence, the primary objective of this study was to evaluate the usability of a device for rehabilitating lip strength and mobility that combines exercises with digital games. Secondary objectives included assessing the influence of age, muscle tension, and number of attempts on performance in adults and children.

## METHOD

This is an observational, cross-sectional study with an analytical approach and a convenience sample, divided into two stages. The first stage involved testing with adults to analyze the instrument's usability, and the second stage involved testing with children treated at the Clinics Hospital of the Federal University of Minas Gerais (UFMG). Both stages constituted a pilot study, and no sample size calculation was performed. The study was approved by UFMG’s Research Ethics Committee under approval number 3,342,534 and CAAE 13318719.9.0000.5149. All adult participants signed an informed consent form, and the children signed an assent form.

**Figure 1:**
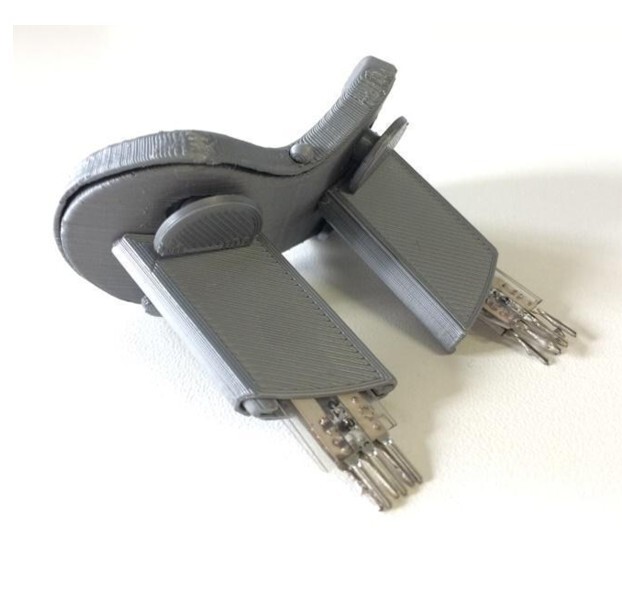
Research instrument

**Figure 2:**
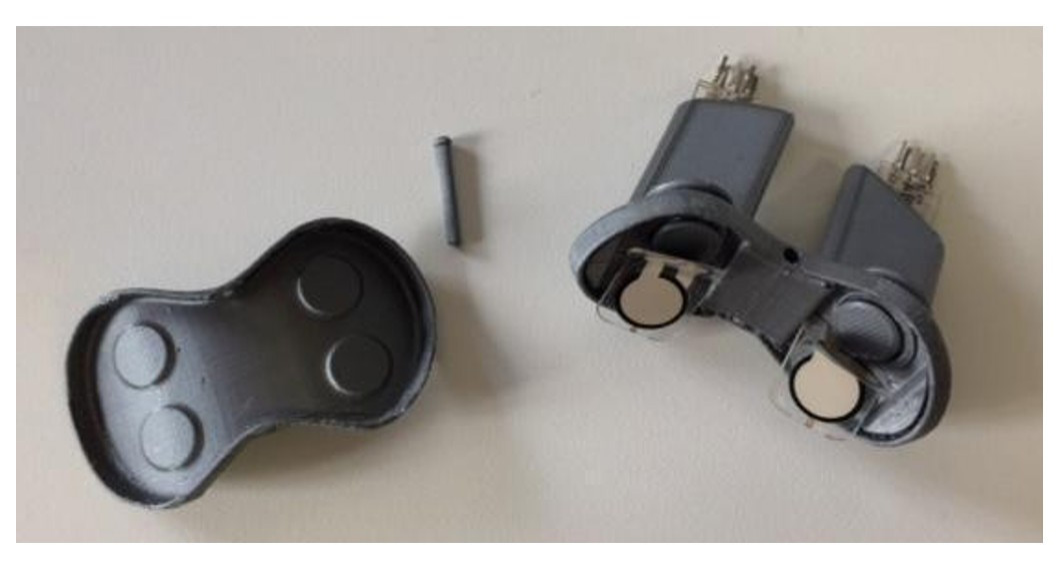
Interior of the instrument, showing the four sensors positioned in each quadrant

**Figure 3:**
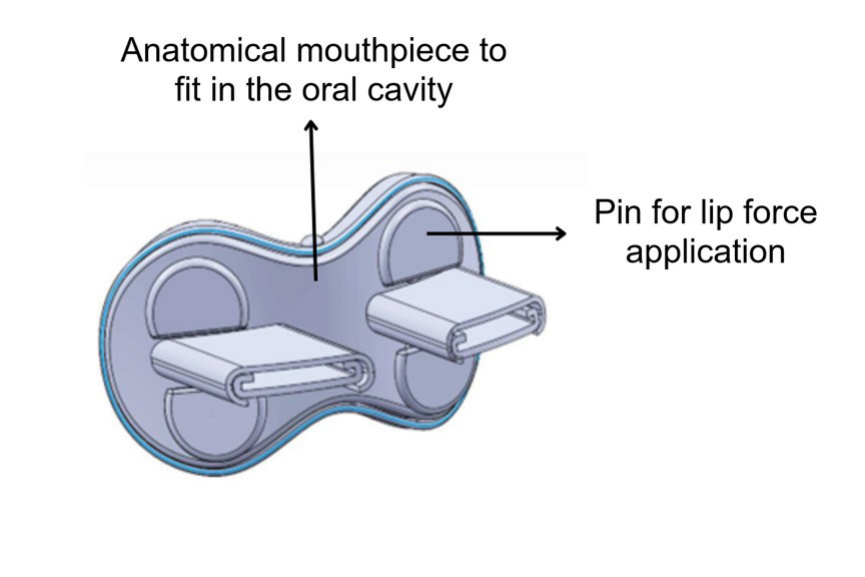
Representative illustration of the research instrument, highlighting the mouthpiece, which fits into the oral cavity, and one of the pins for applying lip force.

The instrument used in the research (Figure 1) consists of a functional anatomical prototype for strength therapy of the orbicularis oris muscle, which serves as a controller for digital games. The instrument has an anatomically designed mouthpiece appropriate for fitting into the oral cavity. The piece has two main parts that fit together and accommodate four Flexiforce force sensors (Tekscan®, Texas, USA), strategically positioned to occupy the four quadrants of the lips (upper right, upper left, lower right, and lower left) (Figure 2). Each sensor has its sensitive area in direct contact with a pin of the same diameter as this sensitive area, so that the four pins transmit the force exerted on the lips to the four sensors (Figure 3).

The instrument allows the user to execute commands with their lips according to the game's proposed activities. The commands' force is captured by sensors, which measure the forces of the upper and lower orbicularis oris and the left and right sides of each lip separately.

The sensors are pressed when the user moves their lips toward their teeth with the instrument in the oral cavity. When pressed by the lips, the sensors produce analog voltage signals that are processed, transmitted, and stored digitally, converting them to force (in newtons) using equations generated during calibration. The analog-to-digital conversion is performed within an ATMEGA328 microprocessor integrated into the Arduino UNO development platform.

Known forces were applied to calibrate each sensor, and the output voltage was equated to these forces. This step was repeated eight times for each known force. Calibration weights were placed on a support with a base of the same diameter as the sensor's sensitive area. Each sensor was calibrated for a force ranging from 0 to 25 N.

**Figure 4:**
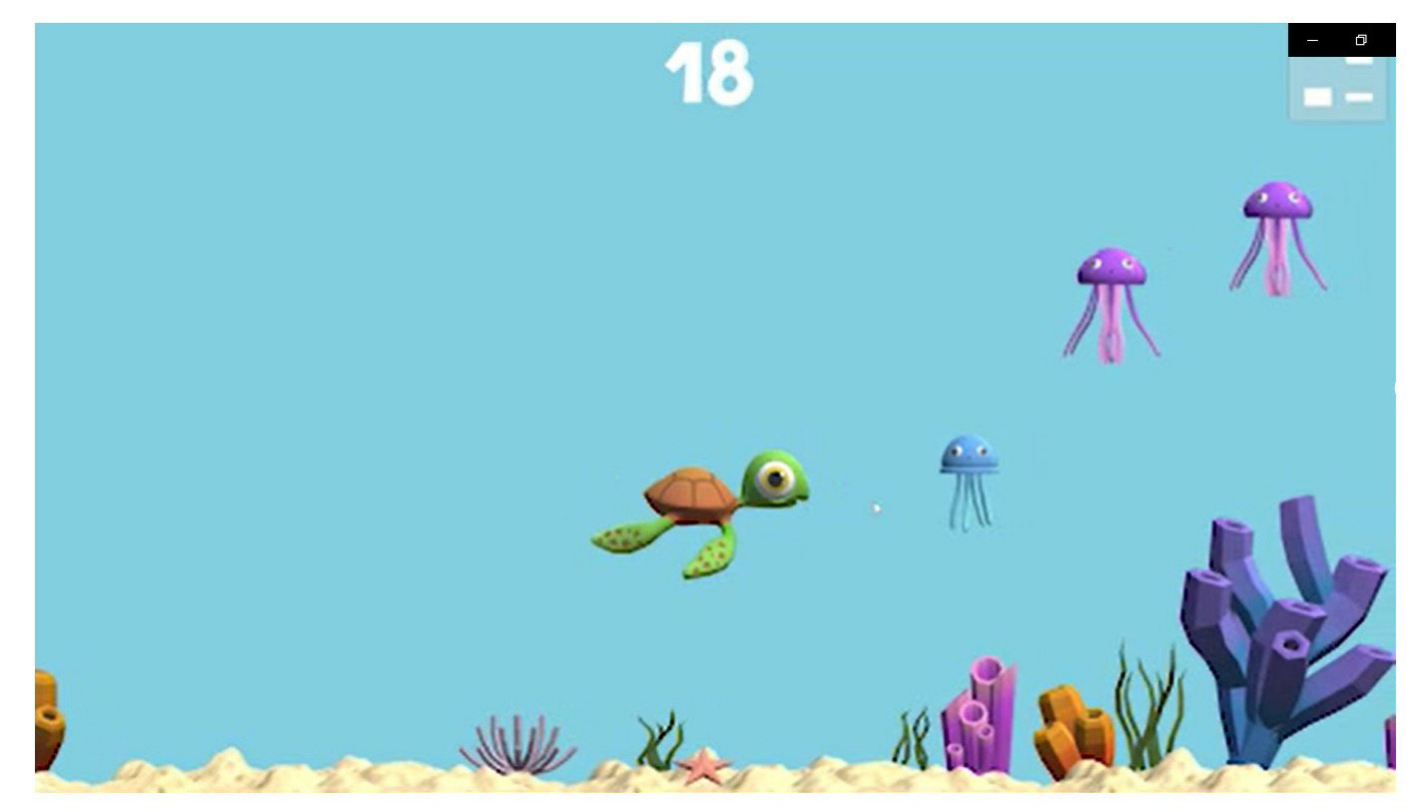
Computer game used in the research

The forces measured by the device are converted into movements in digital games (Figure 4). In the one used in the study, the player must achieve different levels of force (dynamic muscle contraction) and sustain that force for specific periods (static muscle contraction). The game was developed with the theme "Under the Sea," in which the character represented by the user was a turtle, and the goals to be reached were represented by images of small marine animals. When the instrument was pressed, the turtle moved upward with an amplitude proportional to the force applied by the user. To reach the targets at the bottom of the screen (with a lower level of difficulty), the player decreased the applied force so that the turtle remained at the bottom of the screen. To reach higher targets (with a higher level of difficulty), they increased the force applied with the lips to the instrument so that the turtle rose to higher regions of the screen. For each target reached, the user received a score, which varied according to the target's difficulty level, proportional to the level of force applied with the lips.

The force required to score a point was based on the maximum voluntary contraction. This was achieved by placing the instrument in the participant's oral cavity and asking the participant to simultaneously squeeze the four pins with their lips, using a counter-resistance lip movement toward the teeth (the same movement required during the game) with the maximum force possible. The procedure was repeated three times, and the instrument used the maximum value as a reference.

The game presented targets that required the user to exert 15%, 30%, and 45% of their maximum lip force, corresponding to difficulty levels 1, 2, and 3, respectively. Achieving targets of difficulty level 1 increased the score by 2 units, targets of difficulty level 2 increased it by 5 units, and targets of difficulty level 3 increased it by 10 units.

The values of 15%, 30%, and 45% of each participant's maximum strength were defined to ensure that the activity did not cause excessive muscle fatigue, preserving their ability to interact during the game. We avoided excessively high effort levels, which could demotivate participants and compromise the game experience, thus balancing the physical demands necessary for muscle training with engagement. The adequacy of the strength levels defined for different age groups is ensured by the process of achieving maximum individual voluntary contraction, as the force required to reach each target was established based on individual maximum capacity.

The game setup was adjusted for the participant to perform isotonic movements (dynamic muscle contraction) in the first few seconds of the game. Thus, the game began at rest, and then targets of difficulty levels 1, 2, 3, 2, 1, 0, appeared in sequence, forming a zigzag movement, forcing the character to climb up and down to reach the targets. This sequence was repeated five times. The participant then had 6 seconds of rest and then began repeating 10 targets of the same level in sequence, starting with level 1, moving on to levels 2 and 3 (static muscle contraction). Thus, they could reach a maximum score of 290 points if they managed to reach all targets.

### Tests with adults

The study included 11 adults (six females and five males), aged 20 to 41 years, with a mean of 25.3 years and a standard deviation of 7.4 years. The Inclusion criteria were participants aged 18 to 60 years, with all central and lateral incisors, no dental malocclusions that could interfere with the instrument's fit in the oral cavity, no changes in any of the MBGR Orofacial Myofunctional Assessment Protocol lip tests^(24)^, no cognitive or visual impairments that could alter test performance, and no pervasive developmental disorders. The exclusion criteria for these participants were not having consented to the lip assessment, lacking interrater agreement in the lip tension assessment, and not having completed any of the three game trials or the usability evaluation questionnaire.

Two previously trained and calibrated speech-language-hearing evaluators with experience in the treatment of orofacial myofunctional disorders performed the lip assessment with the MBGR protocol lip tests^(24)^. The study included only adults whose lip tension assessment had an agreement between the evaluators. They evaluated the usual lip position, upper lip shape and length, appearance of the upper lip frenulum and mucosa, and lip tone/tension.

Lip tone/tension was assessed using a counter-resistance test and observation of habitual lip position. The participant was positioned facing the evaluator, seated with their back and head supported by the chair back. After receiving instructions on the procedure, the evaluator inserted a gloved finger into the lower oral cavity, near the central incisors, and applied traction in an anterior direction. The participant was asked to pull the evaluator's finger toward their own teeth. The procedure was repeated for the upper lip, observing the participant's ability to maintain muscle contraction for 10 seconds. Participants were considered to have adequate tone/tension if they presented lip closure in their habitual posture and had no difficulty maintaining muscle contraction for 10 seconds during the counter-resistance test. Participants were classified as having decreased tone/tension when the counter-resistance test had abnormal results, such as difficulty sustaining the force for 10 seconds and tremor or absence of lip closure in their habitual posture. Participation in the study was limited to participants who achieved agreement in both examiners’ assessments.

The instrument was then presented to the participants, sanitized with 70% alcohol, wrapped in transparent polyvinyl chloride (PVC), and inserted into the oral cavity. Participants sat with their feet and backs supported, facing the computer screen. Once seated, they were instructed on the lip movements required to play the game and completed a 20-second training session, in which they practiced the game by executing the required movements, without scoring any points.

After training, they performed three attempts at the game, with a 2-minute interval between each attempt. The three attempts had the same target structure and were identical in terms of target position, force required to hit each target, timing of target appearance, and game duration.

Participants were then instructed to complete the System Usability Scale (SUS)^(25)^, validated^(26)^ to assess the instrument's usability. This questionnaire, considered an effective tool for assessing product usability, has 10 questions, with responses given on a Likert scale from 1 to 5, with 1 representing "strongly disagree" and 5 representing "strongly agree." The total score is calculated by adding the scores for each item. The individual score for odd-numbered items^(1,3,5,7,9)^ is the score received minus 1. For even-numbered items^(2,4,6,8,10)^, the contribution is 5 minus the score received. The sum of all scores is then multiplied by 2.5^(26)^. The total score ranges from 0 to 100, with 68 being the cutoff. Therefore, values above 68 indicate good usability^(26,27)^.

### Tests with children

Nine children participated in this phase (five girls and four boys), aged 7 to 12 years (mean age 10.7 years and standard deviation 1.6 years). The children were recruited from HC-UFMG’s Mouth Breathing Outpatient Clinic, and five of them had altered lip tension. The Inclusion criteria were children undergoing speech-language-hearing therapy, aged 7 to 12 years, with all central and lateral incisors, no dental malocclusions that could interfere with the instrument's fit in the oral cavity, no cognitive impairments that could affect test performance, no pervasive developmental disorders, no visual impairments, and no complete lip paralysis. The exclusion criteria were not having consented to lip assessment, lacking examiner agreement on lip tone/tension assessment, and failing to complete any of the three game trials.

Two evaluators performed a qualitative clinical lip assessment (similar to that performed in adults) to classify the children according to lip tone/tension. Only children who obtained agreement between the evaluators’ assessments were included in the study. The assessment was performed through muscle palpation and counter-resistance, in addition to the assessment of habitual lip position, upper lip shape and length, and appearance of the upper lip frenulum and mucosa. This assessment was performed using the MBGR lip assessment items^(24)^. All children with abnormal lip tone/tension were recruited from HC-UFMG’s Mouth Breathing Outpatient Clinic, and children without changes were recruited from HC-UFMG’s Speech-Language-Hearing Outpatient Clinic, where they were receiving various treatments, except for oral motor therapy.

After the clinical evaluation, the game and instrument were presented to the child and their guardian. The instrument was then sanitized with 70% alcohol, wrapped in clear PVC, and inserted into the child's oral cavity. The procedure to obtain maximum voluntary contraction strength was performed three times, followed by a 20-second training session. Finally, the game was played in three identical attempts, with a 2-minute interval, as with the adults. All participants received the same instructions:

*Keep the instrument in your mouth and press these pins* (the pins were shown) *with your lips toward your teeth. The more pressure you apply to the pins, the higher the turtle will rise; the less pressure you apply, the lower it will fall. You will need to grab the little creatures that appear in front of the turtle. If they are high up, you will need to press firmly with your lips, and if they are low down, you will need to reduce the force*.

No influence from parents or guardians was permitted during the children's interaction with the instrument. Parents and guardians were asked to wait in a reserved area while the child remained in the data collection room with the researcher.

### Data analysis

The usability data were analyzed descriptively, by individual values, mean scores, and standard deviations.

The participants’ game scores were the research’s response variable. Explanatory variables included experience with the game (first attempt, second attempt, or third attempt), age group (adults or children), age in the adult group (considering the median as the cutoff), lip tension classification (normal or decreased) in the child group, and the usability assessment score in the adult group.

Data on participants' game scores were presented descriptively, using individual values and the group’s measures of central tendency and dispersion for each trial. The Shapiro-Wilk test was used to assess the distribution of continuous variables, which indicated a normal distribution for most variables. The t-test was used to compare the number of trials between groups (children vs. adults, adults over vs. under 22 years old, and children with normal vs. abnormal tension). The paired-sample t-test was used for intragroup comparisons of scores across trials.

Pearson's correlation coefficient was used to analyze the association between the adults’ attempts and the SUS score, classified as follows: 0–0.2, very poor correlation; 0.21–0.4, poor correlation; 0.41–0.6, fair correlation; 0.61–0.8, good correlation; 0.8–1.0, excellent correlation.

## Results

### Usability analysis

Individual usability scores ranged from 65 to 100, with a mean of 91.1 and a standard deviation of 11.0 (Table 1).

**Table 1:** Assessment of instrument usability by adult participants

Participant	Question 1	Question 2	Question 3	Question 4	Question 5	Question 6	Question 7	Question 8	Question 9	Question 10	Score
1	5	1	5	2	3	2	5	1	4	2	85
2	4	1	4	4	5	1	5	1	5	1	87.5
3	3	2	4	4	4	2	4	2	3	2	65
4	5	1	5	1	4	1	5	1	4	1	95
5	5	1	5	1	5	1	5	1	5	1	100
6	5	1	4	4	5	1	5	1	5	4	82.5
7	5	1	5	1	5	1	5	1	5	1	100
8	5	1	5	1	5	1	5	1	5	1	100
9	5	1	5	1	5	1	5	1	5	1	100
10	5	1	4	4	5	1	5	1	4	1	87.5
11	5	1	5	1	5	1	5	1	5	1	100

Question 1 - I would use this instrument frequently; Question 2 - The instrument is unnecessarily complex; Question 3 - The instrument is easy to use; Question 4 - I need help operating the instrument; Question 5 - The various functions of this instrument are well integrated; Question 6 - There are many inconsistencies in the instrument; Question 7 - People will learn to use the instrument easily; Question 8 - The instrument is too complicated to use; Question 9 - I felt very confident using the instrument; Question 10 - There is a lot of information to learn before using the instrument

### Correlation between SUS score and game score

Pearson's correlation coefficients between SUS scores and the scores obtained in the first, second, and third attempts, and the mean of the attempts were respectively -0.225 (very poor correlation), 0.431 (poor correlation), 0.412 (poor correlation), and 0.336 (very poor correlation).

### Influence of age

Table 2 presents the comparative analysis between the mean scores of adults under and over 22 years old. There was no statistically significant difference between the results.

**Table 2:** Comparative analysis between the mean scores of adults under 22 years old and over 22 years old

Age	Score		p-value*
Under 22 years (n = 5)	Mean	103.8	0.543
	Median	107.3	
	Standard deviation	16.3	
	Minimum	84.0	
	Maximum	121.3	
Over 22 years (n = 4)	Mean	110.5	
	Median	114.0	
	Standard deviation	14.8	
	Minimum	90.7	
	Maximum	123.3	

* Paired t-test

Table 3 presents a comparative analysis between the scores of children with normal lip tension and adults. There was a statistically significant difference when comparing the results of children and adults regarding the first attempt and the mean of the children's attempts, with children obtaining lower scores than adults.

**Table 3:** Comparative analysis between the scores of children with normal lip tension and adults.

Attempt		Adults (n = 9)	Children (n = 4)	p-value*
1st	Mean	105.6	74.3	0.020
	Median	105.0	82.0	
	Standard deviation	17.7	13.3	
	Minimum	69.0	59.0	
	Maximum	129.0	82.0	
2nd	Mean	105.6	74.0	0.055
	Median	105.0	80.0	
	Standard deviation	22.5	28.9	
	Minimum	73.0	34.0	
	Maximum	129.0	102.0	
3rd	Mean	109.2	80.7	0.084
	Median	119.0	70.0	
	Standard deviation	23.2	18.5	
	Minimum	70.0	70.0	
	Maximum	129.0	102.0	
Mean of the attempts	Mean	106.8	75.4	0.004
	Median	107.7	78.8	
	Standard deviation	15.1	12.1	
	Minimum	84.0	58.0	
	Maximum	123.3	86.0	

* T-test

### Influence of lip tension

Table 4 presents the comparative analysis between the scores obtained by children with normal lip tension and those obtained by children with decreased tension. The comparisons did not indicate any statistically significant difference.

**Table 4:** Comparative analysis between the scores obtained by children with normal tension and by children with decreased tension

Lip tension	Characteristics	1st attempt	2nd attempt	3rd attempt	Mean
Normal (n = 4)	Mean	74.3	74.0	80.7	75.4
	Median	82.0	80.0	70.0	78.8
	Standard deviation	13.3	28.9	18.5	12.1
	Minimum	59.0	34.0	70.0	58.0
	Maximum	82.0	102.0	102.0	86.0
Decreased (n = 5)	Mean	76.3	63.3	94.0	75.4
	Median	85.5	54.5	98.0	78.0
	Standard deviation	34.3	34.3	34.7	16.1
	Minimum	26.0	25.0	51.0	55.0
	Maximum	107.0	119.0	129.0	98.7
p-value*		0.927	0.623	0.578	0.995

* T-test

### Influence of the number of attempts

Table 5 presents a comparative analysis of the children’s and adults’ attempts. Although children scored higher on the third attempt, there was no significant difference in the scores obtained by children or adults between the different attempts.

**Table 5:** Comparative analysis between game scores in the different attempts by children and adults

Adults	Attempt	Mean	Standard deviation	p-value*
1st	105.6	17.7	1.000
2nd	105.6	22.5	
1st	105.6	17.7	0.751
3rd	109.2	23.2	
2nd	105.6	22.5	0.558
3rd	109.2	23.2	
Children, regardless of lip tension (n = 9)	1st	64.5	34.0	0.412
	2nd	79.0	39.3	
	1st	64.5	34.0	0.420
	3rd	91.0	36.6	
	2nd	79.0	39.2	0.500
	3rd	91.0	36.6	
Children with normal lip tension	1st	74.3	13.3	0.670
(n = 4)				
	2nd	74.0	28.9	
	1st	74.3	13.3	0.673
	3rd	80.7	18.5	
	2nd	74.0	28.9	0.740
	3rd	80.7	18.5	
Children with decreased lip tension	1st	76.3	34.3	0.532
(n = 5)				
	2nd	63.3	34.3	
	1st	76.3	34.3	0.557
	3rd	94.0	34.7	
	2nd	63.3	34.3	0.392
	3rd	94.0	34.7	

* Paired t-test

## DISCUSSION

### Usability analysis

This research provided preliminary data on the usability of the device for rehabilitating lip strength and mobility combined with digital games. This instrument can elevate speech-language-hearing therapy to a more playful level, potentially promoting greater adherence and motivation, especially among children. It also allows therapists to monitor and record the patient's progress throughout the therapy.

The SUS results indicate that adults' responses to the device's usability were satisfactory, considering the cutoff of 68 points for this questionnaire^(26,27)^. However, there were disagreements on the questions "I need help to operate the instrument" and "I need to learn a lot of information before using the instrument," indicating that the device may not be sufficiently intuitive, and that changes are needed to facilitate its use. Therefore, improvements to the device's design and interface are needed to facilitate use, especially considering children, who may have more difficulty operating it independently.

The mean total test score indicated that the instrument, in the participants' opinion, presented good usability, and that the usability judgment is not related to game performance, given the lack of correlation between the SUS score and the game score. It is worth considering that, although validated and widely used, the SUS has limitations when applied to specific rehabilitation devices, whose ergonomic and functional aspects can significantly influence the perception of usability, and that the instrument's original cutoff may not be the most appropriate for this type of device. No studies were found that used the SUS to evaluate specific lip rehabilitation instruments, but in the context of speech-language-hearing rehabilitation, the score obtained for an application designed for swallowing training, in the perception of older users, presented means of 56.0 and 70.8 points, respectively, for older people with lower and higher educational levels and familiar with using mobile applications^(28)^. Another application, designed for speech training, received a mean score of 89.2 from healthy volunteers without speech impairments^(29)^. The lack of studies using equipment similar to that used in this one makes it difficult to compare SUS scores in the specific context of lip rehabilitation. However, the score obtained in this study (91.1) is much higher than the cutoff and can be considered representative of good usability.

Despite the significant step forward, the sample size of only 11 adults in the usability assessment can be considered a limitation for generalizing the device's usability. Furthermore, the age range of the sample participating in the usability assessment (20 to 41 years old) prevents the data from being generalized to other age groups. Dividing this sample into sexes and younger age groups was unfeasible given its small size. Future research is recommended to analyze users' usability perceptions in greater depth.

It should be noted that the study did not investigate the influence of familiarity with digital games. Such familiarity may have influenced the perception of usability, since the game may be more intuitive for some user profiles. On the other hand, the training provided to all participants may have minimized the effects of prior experience with digital games. Although there was no statistically significant relationship between game performance and the participants' perceived usability, we suggest that future research investigate the time participants spend playing video games per week.

### Influence of age

There was no difference in scores between adults under and over 22 years old. The cutoff of 22 years was established because it is the median age of the adult sample. The literature indicates that differences in performance in tongue-driven computer games may exist when comparing more distant age groups, as in the study by Kothari et al.^(30)^, whose participants aged 21 to 35 years performed better than those aged 52 to 72 years. This difference may be explained by the loss of muscle mass^(31)^ and reduced speed and dexterity^(32,33)^ typical of aging. No similar studies on the lips were found, but research indicates a reduction in lip strength^(34)^ and resistance^(34-36)^ after age 60.

When comparing adults and children with normal tension, the children's mean score was lower. This finding can be explained by the motor development of the perioral muscle morphology and the central nervous system, characterized by increased diameter and myelination of axons during childhood and adolescence^(37)^. A study by Saitoh et al.^(38)^ associates lip strength with hormonal changes that accompany sexual maturation and somatic growth in functional capacity. Thus, children’s structural immaturity should be considered to explain the difference in performance^(38)^. Central nervous system maturation also encompasses the development of cognitive and visuomotor skills related to making predictions about the occurrence and duration of future events and programming reaction time^(39)^, skills required in games.

It was also found that the instrument size must be considered in the analysis of the applied force^(38)^. The volume of the instrument used in this study may have influenced the scores of children, who have smaller lips than adults. Therefore, the instrument dimensions must be adapted to children.

This study did not investigate the influence of participant sex on the results, especially due to the small sample size for sex subdivision. However, studies with a language rehabilitation instrument using digital games found no sex differences for adults^(12)^ or children^(10)^. The literature attributes better performance in digital games to males, justified by the greater time typically spent on this activity^(30)^. However, the instrument used in this study has an atypical functioning for all participants, not dependent on manual dexterity, which minimizes the influence of familiarity.

### Influence of lip tension

Tests conducted with children indicated that the instrument is promising and can be used for this population. However, given the few individuals in the samples, the analysis of the results should be interpreted with caution, especially when separated according to abnormal lip tension. Children and adults had similar mean scores when comparing the first and second attempts, with a small increase in score on the third attempt. However, the results of children with abnormal lip tension did not follow this pattern. Their performance worsened on the second attempt and improved on the third. Further investigation is needed to understand the interaction between the effects of fatigue and motor learning in this population. The force required in the game was calculated individually at the time of calibration, considering each participant’s maximum force, which may explain the lack of difference between adults of different age groups and between children with normal and decreased tension, since the game required a lower level of force from those with decreased tension.

### Influence of the number of attempts

Despite the lack of significant difference between the scores obtained in each attempt, both adults and children had higher scores on the third attempt, corroborating other studies^(12,40,41)^ involving computer games associated with orofacial muscle-activated devices. These studies^(12,40,41)^ associated the increase in scores on successive attempts with a learning effect. According to Huo and Ghovanloo (2010), individuals gain experience quickly in this type of activity and improve their performance even with few attempts^(40)^. Since the study had only three attempts, it is suggested that future research explore in more depth how this learning effect occurs over several attempts and whether there is a stabilization point in performance.

It is interesting to mention that the training carried out before the first attempt was important to minimize a possible lack of understanding by the participants about how to act in the game dynamics or how to perform the movement with their lips. This could have exacerbated the effect of the training if the first contact with the game had occurred on the first attempt.

Contrary to what was observed, it was expected that children's attention and motivation would decrease over the three attempts, given that the game was identical across all of them, and that muscle fatigue could negatively impact participants' performance, especially in children with decreased muscle tension. However, these conditions, if present, were not enough to worsen participants' performance, as the third attempt yielded the best performance across all study groups.

### Limitations and suggestions for future research

The study is innovative and offers promising insights into the use of digital games for orofacial rehabilitation. One limitation of the study is its sample size, particularly regarding children with abnormal lip tension. Other important limitations include the lack of quantitative measurements of strength, contraction duration, and muscle fatigue. Such measurements would be important for a more detailed assessment of the device's effectiveness and for comparison with other therapy methods. Thus, the lack of quantitative data limits the understanding of the device's physiological benefits in terms of muscle rehabilitation and indicates the need for further research to fill this gap. The need for adaptation to different population groups also reduces the validity and immediate applicability of the results. Another limitation of the study concerns the differentiated scoring for targets with varying levels of difficulty. This was a device implemented to increase the reward for targets requiring greater effort, but it hindered comparative analysis of performance among users. In this sense, more detailed reports, including information on the number of targets of each difficulty level achieved during the game, should be incorporated into the software processing for more robust feedback on each user's performance.

Finally, the lack of information about the participants' familiarity with digital games can also be cited as a limitation. It is worth noting that the instrument used has its own characteristics and an unusual operation, not dependent on manual dexterity, which minimizes the influence of familiarity with traditional video game joysticks.

It is suggested that future research use the method with individuals with varying degrees of alteration in lip tone/tension, across different ages, and with diverse clinical conditions, such as facial paralysis and Down syndrome, with targets at different strength levels. It is also suggested that quantitative measures of muscle strength and fatigue be incorporated. Furthermore, longitudinal studies should compare this instrument with traditional speech-language-hearing therapy methods and use objective methods to measure children's enjoyment and interest.

## CONCLUSION

The evaluated instrument demonstrated good usability in the opinion of the adults who tested the equipment. The correlation between the adults' game scores and their opinion of usability was poor or very poor, highlighting the lack of a relationship between difficulty in playing and success in completing the tasks. There was no statistically significant difference in game scores between adults over and under 22 years old. Moreover, children scored lower than adults on the first attempt and on the mean of the attempts. There was no statistically significant difference between the scores of children with normal and abnormal lip tension. There was no significant difference between the children’s and adults’ scores across the different attempts.
