# Supplementary material for: Usability of a device for lip strength and mobility rehabilitation associated with digital games: a pilot study
Source: Codas. 2025 Oct 20;37(5):e20240271. doi: 10.1590/2317-1782/e20240271en (PMC12617285; doi:10.1590/2317-1782/e20240271en)
Supplement: Translation

# Translation: Usabilidade de um dispositivo para reabilitação da força e mobilidade dos lábios associado a jogos digitais: estudo piloto

**DOI:** 10.1590/2317-1782/e20240271pt

**Authors:** Joyce Marques Barroso, Clarice Magnani Figueiredo, Eduardo Pena Castro Fantini, Marcos Antônio Abdalla, Andréa Rodrigues Motta, Estevam Barbosa Las Casas, Renata Maria Moreira Moraes Furlan

**Affiliations:** 1 Programa de Pós-graduação em Ciências Fonoaudiológicas, Universidade Federal de Minas Gerais – UFMG - Belo Horizonte (MG), Brasil.; 2 Programa de Pós-graduação em Engenharia de Estruturas, Universidade Federal de Minas Gerais – UFMG - Belo Horizonte (MG), Brasil.; 3 Wildlife Studios - São Paulo (SP), Brasil.; 4 Serviço Nacional de Aprendizagem Industrial - SENAI SAT - São João Del Rei (MG), Brasil.; 5 Departamento de Fonoaudiologia, Universidade Federal de Minas Gerais – UFMG - Belo Horizonte (MG), Brasil.; 6 Departamento de Engenharia de Estruturas, Universidade Federal de Minas Gerais – UFMG - Belo Horizonte (MG), Brasil.

**Keywords:** Força Muscular, Jogos de Vídeo, Reabilitação, Lábios, Terapia Miofuncional

## Abstract

**Objetivo:**

Avaliar a usabilidade de um dispositivo para reabilitação da força e da mobilidade dos lábios que associa exercícios a jogos digitais e verificar a influência da idade, da tensão muscular e do número de tentativas no desempenho de adultos e crianças.

**Método:**

Estudo observacional, transversal, do qual participaram 11 adultos e nove crianças. Os participantes utilizaram um instrumento que consiste em um protótipo anatômico funcional para reabilitação do músculo orbicular da boca, que funciona como comando para jogos digitais e é acionado pelo movimento de contrarresistência realizado pelos lábios. Os participantes executaram um jogo com o dispositivo e receberam uma pontuação proporcional ao seu desempenho. Após, os participantes adultos responderam o questionário System Usability Scale (SUS) para avaliação da usabilidade do instrumento. A pontuação dos participantes foi comparada considerando idade, tensão muscular dos lábios e número de tentativas.

**Resultados:**

A usabilidade foi considerada boa, com média de 91,1 e desvio-padrão de 11 pontos. Não houve diferença entre as pontuações obtidas nas diferentes tentativas, bem como quando comparadas as pontuações dos adultos menores e maiores de 22 anos, ou quando comparadas crianças com tensão de lábios normal e diminuída. Na comparação da pontuação entre adultos e crianças, houve diferença com relevância estatística para a primeira tentativa e para a média das tentativas.

**Conclusão:**

O instrumento apresentou boa usabilidade, com influência da faixa etária (adultos x crianças) na pontuação dos participantes.

## INTRODUÇÃO

Os avanços tecnológicos, no que se refere às Ciências da Saúde e, especificamente, à Fonoaudiologia, permitem o aprimoramento da avaliação e das estratégias terapêuticas utilizadas durante intervenção para reabilitação da musculatura do sistema estomatognático. Uma dessas estratégias que pode ser utilizada com a função de promover melhor interação dos pacientes é a gameterapia^(1,2)^.

A gameterapia utiliza os “*Serious Games*”, uma categoria de jogos desenvolvida não apenas para entretenimento de seus usuários, mas também com o propósito de intervenção e reabilitação de suas possíveis alterações^(3)^. O objetivo dos “*serious games*” é promover atividades mais lúdicas e atrativas, ao mesmo tempo voltadas ao aprendizado e ao treinamento, e para o desenvolvimento de habilidades específicas^(2)^. Alguns dos seus elementos fundamentais são o estímulo das funções cognitivas, a motivação e as possibilidades de construção de novos conhecimentos^(4)^.

O uso de jogos digitais associado à mioterapia pode proporcionar melhor adesão dos pacientes^(5)^ e promover mudanças, não apenas a nível muscular, como também no córtex motor^(6)^. Além disso, esse tipo de inovação pode elevar a qualidade dos atendimentos, pois diverte e entretém os utilizadores, principalmente as crianças^(7)^. Por isso, estão sendo cada vez mais explorados nos programas de treinamento motor^(8,9)^.

Na prática da Motricidade Orofacial, jogos computacionais associados a exercícios para língua foram desenvolvidos e apresentados em diferentes pesquisas^(10-13)^. Contudo, não foram encontrados estudos que associassem jogos computacionais à reabilitação labial. Os lábios são responsáveis por funções importantes para o nosso corpo, e a alteração de tônus/tensão dessa musculatura dificulta a realização das funções de mastigação, deglutição e fala^(14)^. A hipotonia labial é comum em condições clínicas como a respiração oral^(4)^, paralisia facial^(15)^, má-oclusão dentária^(16)^, doenças neurodegenerativas^(17,18)^ e disfagia^(19)^ e, quando isso ocorre, é necessário o treinamento muscular para ganho de força^(20)^.

Recentemente, foi desenvolvido no Brasil um dispositivo para reabilitação da força e da mobilidade dos lábios^(21)^ que associa os exercícios mioterápicos a jogos digitais. O dispositivo é composto por um instrumento que serve como um console para jogos digitais. Com esse instrumento, o usuário pode executar comandos com os lábios de acordo com as demandas do jogo, que são ajustadas previamente pelo terapeuta^(21).^

Contudo, o dispositivo não foi avaliado quanto à sua usabilidade, que, segundo a *International Organization for Standardization (ISO)* ^(22)^ está relacionada à eficiência, eficácia e satisfação de como um indivíduo interage com o produto para atingir objetivos específicos. Os principais métodos de avaliação da usabilidade utilizam dados dos usuários ou de especialistas na área^(23)^. A avaliação da usabilidade permite verificar se o sistema atende à necessidade real do utente, identificar falhas e possibilitar a correção, oferecendo uma melhor experiência ao usuário.

Diante do exposto, o objetivo primário do presente estudo foi avaliar a usabilidade do dispositivo para reabilitação da força e da mobilidade de lábios que associa exercícios a jogos digitais. Como objetivos secundários buscou-se verificar a influência da idade, da tensão muscular e do número de tentativas no desempenho de adultos e crianças.

## MÉTODO

Trata-se de um estudo observacional, transversal, com abordagem analítica e amostra de conveniência, dividido em duas etapas. A primeira etapa referiu-se a testes com adultos para análise da usabilidade do instrumento e a segunda etapa consistiu em testes realizados com crianças em atendimento no Hospital das Clínicas da Universidade Federal de Minas Gerais (UFMG). Ambas as etapas constituíram um estudo piloto, não sendo realizado cálculo amostral. O estudo foi aprovado pelo Comitê de Ética em Pesquisa da Universidade Federal de Minas Gerais sob o número de parecer 3.342.534 e CAAE 13318719.9.0000.5149. Todos os participantes adultos assinaram o Termo de Consentimento Livre e Esclarecido e as crianças o Termo de Assentimento.

O instrumento utilizado na pesquisa (Figura 1) consiste em um protótipo anatômico funcional para terapia de força do músculo orbicular da boca, que serve como controlador para jogos digitais. O instrumento é constituído de uma peça com anatomia apropriada para encaixe no vestíbulo oral. A peça é composta por duas partes principais que se encaixam e acomodam quatro sensores de força Flexiforce (marca Tekscan®, Texas, Estados Unidos) em seu interior, posicionados estrategicamente de maneira a ocupar os quatro quadrantes dos lábios (superior direito, superior esquerdo, inferior direito e inferior esquerdo) (Figura 2). Cada sensor tem sua área sensível em contato direto com um pino de mesmo diâmetro dessa área sensível, de forma que os quatros pinos transmitem a força realizada nos lábios para os quatro sensores (Figura 3).

**Figura 1:** Instrumento da pesquisa

**Figura 2:** Interior do instrumento, mostrando os quatro sensores posicionados em cada quadrante

**Figura 3:** Ilustração representativa do instrumento de pesquisa com destaque para a peça principal, que se encaixa no vestíbulo oral e de um dos pinos de aplicação da força labial

O instrumento permite que o usuário execute comandos com os lábios de acordo com as atividades propostas no jogo. Os comandos têm sua força captada a partir dos sensores, que permitem medir separadamente as forças do orbicular da boca superior e inferior, bem como dos lados esquerdo e direito de cada lábio.

Os sensores são pressionados quando o usuário, após encaixar o instrumento no vestíbulo oral, realiza o movimento dos lábios em direção aos dentes. Ao serem pressionados pelos lábios, os sensores produzem sinais analógicos em tensão que são tratados, transmitidos, processados e armazenados em meio digital e convertidos para força (em newtons) por meio das equações geradas na calibração. A conversão analógico-digital é executada dentro de um microprocessador modelo ATMEGA328, integrado na plataforma de desenvolvimento Arduino UNO.

Para calibrar cada sensor, foram aplicadas forças conhecidas, e equiparou-se o valor de saída, em tensão, com estas forças. Este passo foi repetido oito vezes para cada força conhecida. Utilizou-se um suporte com base de igual diâmetro à área sensível do sensor, sobre o qual foram posicionados pesos de calibração. Cada sensor foi calibrado para um faixa de força entre 0 e 25 N.

As forças mensuradas pelo aparelho são convertidas em movimentos nos jogos digitais (Figura 4). Foi utilizado um jogo em que o usuário, além de alcançar diferentes níveis de força (contração muscular dinâmica), também precisava sustentar a força por determinados períodos (contração muscular estática). O jogo foi desenvolvido com o tema “Fundo do Mar”, no qual o personagem representado pelo usuário era uma tartaruga e os alvos a serem alcançados foram representados por imagens de pequenos animais marinhos. Quando o instrumento era pressionado, a tartaruga movimentava-se para cima com amplitude proporcional à força aplicada pelo usuário. Para que fosse possível alcançar os alvos na parte inferior da tela (alvos com menor nível de dificuldade), era necessário diminuir a força aplicada para que a tartaruga se mantivesse na parte inferior da tela. Para alcançar alvos mais altos (alvos com maior nível de dificuldade), era necessário aumentar a força aplicada com os lábios no instrumento para que a tartaruga subisse para regiões mais altas da tela. Para cada alvo atingido, o usuário recebia uma pontuação, que variava conforme o nível de dificuldade do alvo, o qual era proporcional ao nível de força a ser realizada com os lábios.

**Figura 4:** Jogo computacional utilizado na pesquisa

O nível da força solicitada ao usuário (necessária para pontuar) foi estipulado a partir da máxima contração voluntária. Para a obtenção da máxima contração voluntária, o instrumento foi posicionado na cavidade oral do participante, o qual foi solicitado a apertar simultaneamente os quatro pinos com os lábios, por meio de um movimento de contrarresistência dos lábios em direção aos dentes (mesmo movimento requerido durante o jogo) com a máxima força possível. O procedimento foi repetido por três vezes e o instrumento considerou o valor máximo como referência.

O jogo utilizado apresentava alvos que requeriam do usuário a realização de 15%, 30% e 45% da sua força máxima labial, referentes aos níveis de dificuldade 1, 2 e 3, respectivamente para alcançá-los. A pontuação aumentava em duas unidades para alvos de nível de dificuldade 1, em cinco unidades por alvo de dificuldade 2 e em dez unidades por alvo de dificuldade 3.

Os valores de 15%, 30% e 45% da força máxima de cada participante foram definidos de forma a garantir que a atividade não causasse fadiga muscular excessiva, preservando a capacidade de interação durante o jogo. Buscou-se evitar níveis de esforço que fossem excessivamente altos, o que poderia desmotivar os participantes e comprometer a experiência do jogo e equilibrar a exigência física, necessária à atividade de treinamento muscular, e o engajamento. A adequação dos níveis de força definidos para diferentes faixas etárias é garantida pelo processo de obtenção da máxima contração voluntária individual, pois a força exigida para alcançar cada alvo foi estabelecida a partir da capacidade máxima individual.

A configuração do jogo foi ajustada para que, nos primeiros segundos do jogo, o participante executasse movimentos de isotonia (contração muscular dinâmica). Sendo assim, o jogo iniciava no repouso e, a seguir, surgiam alvos de nível de dificuldade 1, 2, 3, 2, 1, 0, respectivamente, formando um movimento de zigue-zague e fazendo com que o personagem subisse e descesse para alcançar os alvos. Essa sequência era realizada cinco vezes. Em seguida, o participante tinha seis segundos de repouso e, então, iniciava a repetição de 10 alvos de mesmo nível em sequência, começando pelo nível 1, seguindo para os níveis 2 e 3 (contração muscular estática). Dessa forma, a máxima pontuação que um participante poderia receber, caso conseguisse alcançar todos os alvos, era de 290 pontos.

### Testes com adultos

Foram incluídos 11 adultos, sendo seis do sexo feminino e cinco do sexo masculino, com idades entre 20 e 41 anos, média de 25,3 anos e desvio padrão de 7,4 anos. Como critérios de inclusão, os participantes deveriam ter idades entre 18 e 60 anos; apresentar todos os dentes incisivos centrais e laterais; não apresentar más-oclusões dentárias que pudessem interferir no encaixe do instrumento na cavidade oral; não apresentar alteração em qualquer uma das provas de lábios do Protocolo MBGR^(24)^; não apresentar comprometimentos cognitivos ou visuais que pudessem alterar a realização do teste; ou transtornos invasivos do desenvolvimento. Os critérios de exclusão para estes participantes foram: não ter permitido a realização da avaliação dos lábios; ausência de concordância interavaliador na avaliação da tensão labial; não ter realizado alguma das três tentativas do jogo; ou não ter respondido o questionário de avaliação da usabilidade.

A avaliação dos lábios, realizada por meio das provas de lábios do protocolo MBGR – Avaliação Miofuncional Orofacial^(24)^ foi conduzida por dois avaliadores fonoaudiólogos com experiência no tratamento dos distúrbios miofuncionais orofaciais, previamente treinados e calibrados, sendo incluídos no estudo apenas os adultos que obtiveram concordância na avaliação da tensão labial de ambos os avaliadores. Foram avaliados posição habitual dos lábios, forma, comprimento do lábio superior, aspecto da mucosa e frênulo do lábio superior e tônus/tensão labial.

A avaliação do tônus/tensão labial foi realizada por meio da prova de contrarresistência e da observação da posição habitual dos lábios. O participante foi posicionado de frente para o avaliador, sentado com as costas e a cabeça apoiadas no encosto da cadeira. Após instruções sobre o procedimento, o avaliador inseriu o dedo enluvado no vestíbulo oral inferior, próximo aos dentes incisivos centrais e realizou tração em direção anterior, sendo o participante solicitado a tracionar o dedo do avaliador em direção aos próprios dentes. O procedimento foi repetido para o lábio superior. Foi observada a capacidade do participante de manter a contração muscular por 10 segundos. Foram considerados com tônus/tensão adequada os participantes que apresentavam vedamento labial na postura habitual e não apresentaram dificuldades em manter a contração muscular por 10 segundos na prova de contrarresistência. Os participantes classificados com tônus/tensão diminuída foram aqueles que apresentaram alterações durante a prova de contrarresistência, como dificuldades de sustentar a força por 10 segundos e tremor ou ausência de vedamento labial na postura habitual. A participação no estudo foi limitada aos participantes que alcançaram concordância nas avaliações de ambos os examinadores.

Em seguida, o instrumento foi apresentado aos participantes, higienizado com álcool 70%, envolto com policloreto de vinil (PVC) transparente e inserido na cavidade oral. Os participantes estavam posicionados sentados com os pés e as costas apoiados, de frente para a tela do computador. Após posicionados, eles foram instruídos quanto aos movimentos de lábios necessários para realização do jogo e realizaram um treinamento, com duração de 20 segundos, em que praticaram o jogo executando os movimentos necessários, mas sem qualquer tipo de pontuação associada às ações realizadas.

Após o treinamento, foram realizadas três tentativas do jogo com intervalo de dois minutos entre cada uma delas. As três tentativas apresentavam a mesma constituição de alvos, sendo, portanto, idênticas no que se refere à posição dos alvos, força requerida para atingir cada alvo, momento de aparecimento dos alvos no jogo, bem como duração do jogo.

Em seguida, os participantes foram instruídos a responderem o questionário *System Usability Scale (SUS)* ^(25)^, validado^(26)^, para avaliação da usabilidade do instrumento. Este questionário é considerado uma ferramenta eficaz na avaliação da usabilidade de produtos. É composto por 10 perguntas, onde as respostas são referidas na escala *Likert* de 1 a 5, em que 1 representa discordo fortemente e 5 concordo fortemente. O cálculo para definir a pontuação total é realizado por meio da somatória do escore de cada item. Para os itens ímpares (1, 3, 5, 7 e 9), o escore individual é a nota recebida menos 1. Para os itens pares (2, 4, 6, 8 e 10), a contribuição é 5 menos a nota recebida. Na sequência multiplica-se a soma de todos os escores por 2,5^(26)^. A pontuação total varia de 0 a 100, sendo 68 o ponto de corte. Dessa forma, valores acima de 68 indicam boa usabilidade^(26,27)^.

### Testes com crianças

Participaram desta etapa nove crianças, sendo cinco meninas e quatro meninos, de 7 a 12 anos, média de 10,7 e desvio padrão de 1,6 anos. As crianças foram recrutadas no Ambulatório do Respirador Oral do HC-UFMG e destas, cinco apresentaram alteração na tensão dos lábios. Como critério de inclusão, as crianças deveriam estar em atendimento fonoaudiológico, ter idade entre 7 e 12 anos, apresentar todos os dentes incisivos centrais e laterais, não apresentar más-oclusões dentárias que pudessem interferir no encaixe do instrumento na cavidade oral, comprometimentos cognitivos que pudessem alterar a realização do teste ou transtornos invasivos do desenvolvimento, comprometimentos visuais ou paralisia completa dos lábios. Constituíram critérios de exclusão: não ter permitido a realização da avaliação dos lábios, não apresentar concordância dos examinadores na avaliação do tônus/tensão labial ou não ter realizado alguma das três tentativas do jogo.

Para classificar as crianças conforme o tônus/tensão de lábios, foi realizada uma avaliação clínica qualitativa dos lábios por dois avaliadores, de forma semelhante à realizada nos adultos, sendo incluídas no estudo apenas as crianças que obtiveram concordância na avaliação de ambos. A avaliação foi realizada por meio de palpação do músculo e contrarresistência, além da avaliação de posição habitual dos lábios, forma, comprimento do lábio superior, aspecto da mucosa e frênulo do lábio superior. Esta avaliação foi realizada por meio dos itens de avaliação de lábios do protocolo MBGR – Avaliação Miofuncional Orofacial^(24)^. Todas as crianças que apresentaram alteração do tônus/tensão de lábios foram recrutadas no Ambulatório do Respirador Oral do HC-UFMG e as crianças sem alteração foram recrutadas no Ambulatório de Fonoaudiologia do HC-UFMG, onde se encontravam em atendimentos diversos, com exceção da especialidade de Motricidade Orofacial.

Após a avaliação clínica, foram apresentados o jogo e o instrumento para a criança e seu responsável. Em seguida, o instrumento foi higienizado com álcool 70%, envolto com PVC transparente e inserido na cavidade oral da criança. Foi realizado o procedimento de obtenção da força na máxima contração voluntária, três vezes, seguido de um treinamento de 20 segundos de duração e, finalmente, o jogo foi realizado em três tentativas idênticas, com dois minutos de intervalo, da mesma forma como foi procedido com os adultos. As instruções fornecidas foram as mesmas para todos os participantes:

*mantenha o instrumento encaixado na boca e pressione estes pinos* (mostravam-se os pinos) *com os lábios em direção aos seus dentes, sendo que quanto mais força você fizer nos pinos mais alto a tartaruga subirá, se diminuir a força a tartaruga descerá. Você terá que pegar os bichinhos que aparecem à frente da tartaruga. Se eles estiverem no alto, precisará apertar forte com os lábios, e se estiverem baixos precisará diminuir a força*.

Não foi permitido nenhum tipo de influência dos pais ou responsáveis durante a interação das crianças com o instrumento, sendo solicitado aos pais ou responsáveis que aguardassem em uma área reservada, enquanto a criança permanecia na sala de coleta de dados com o pesquisador.

### Análise dos dados

Os dados de usabilidade foram analisados de maneira descritiva, pelos valores individuais, pontuação média e desvio padrão.

A pontuação obtida pelos participantes no jogo constituiu variável resposta desta pesquisa. Constituíram variáveis explicativas: a experiência com o jogo (1ª tentativa, 2ª tentativa ou 3ª tentativa); faixa etária (adultos ou crianças); idade no grupo de adultos (considerando a mediana como ponto de corte); classificação da tensão labial (normal ou diminuída) no grupo de crianças; e a pontuação na avaliação da usabilidade no grupo de adultos.

Os dados referentes à pontuação dos participantes no jogo foram apresentados, de maneira descritiva, pelos valores individuais e pelas medidas de tendência central e dispersão do grupo, para cada tentativa. Utilizou-se o *teste Shapiro-Wilk* com a finalidade de avaliar a distribuição das variáveis contínuas, que indicou distribuição normal para a maioria das variáveis. Utilizou-se o *teste T* para as comparações do número de tentativas entre os grupos: criança x adulto, adultos com mais ou menos de 22 anos, e criança com tensão normal x criança com tensão diminuída. Utilizou-se o *teste T para amostras pareadas* para as comparações intragrupos das pontuações nas diferentes tentativas.

Para análise de associação entre as tentativas realizadas pelos adultos e a pontuação do SUS, utilizou-se o coeficiente de correlação de Pearson. Para classificar a correlação, utilizou-se a seguinte escala: 0 – 0,2 correlação péssima; 0,21 – 0,4 correlação ruim; 0,41 – 0,6 correlação regular; 0,61 – 0,8 correlação boa; 0,8 – 1,0 correlação ótima.

## RESULTADOS

### Análise da usabilidade

As pontuações individuais para usabilidade variaram de 65 a 100, com média de 91,1 e desvio padrão de 11,0 (Tabela 1).

**Tabela 1:** Avaliação da usabilidade do instrumento pelos participantes adultos

Participante	Questão 1	Questão 2	Questão 3	Questão 4	Questão 5	Questão 6	Questão 7	Questão 8	Questão 9	Questão 10	Pontuação
1	5	1	5	2	3	2	5	1	4	2	85
2	4	1	4	4	5	1	5	1	5	1	87,5
3	3	2	4	4	4	2	4	2	3	2	65
4	5	1	5	1	4	1	5	1	4	1	95
5	5	1	5	1	5	1	5	1	5	1	100
6	5	1	4	4	5	1	5	1	5	4	82,5
7	5	1	5	1	5	1	5	1	5	1	100
8	5	1	5	1	5	1	5	1	5	1	100
9	5	1	5	1	5	1	5	1	5	1	100
10	5	1	4	4	5	1	5	1	4	1	87,5
11	5	1	5	1	5	1	5	1	5	1	100

Questão 1 - Eu utilizaria esse instrumento com frequência; Questão 2 - O instrumento é desnecessariamente complexo; Questão 3 - O instrumento é fácil de usar; Questão 4 - Preciso de ajuda para operar o instrumento; Questão 5 - As diversas funções desse instrumento foram bem integradas; Questão 6 - Existem muitas inconsistências no instrumento; Questão 7 – As pessoas aprenderão a usar o instrumento facilmente; Questão 8 - O instrumento é muito complicado de usar; Questão 9 - Eu me senti muito confiante em utilizar o instrumento; Questão 10 - É preciso aprender muitas informações antes de usar o instrumento

### Correlação entre escore do SUS e pontuação no jogo

Os coeficientes de correlação de Pearson para as correlações do escore do SUS com a pontuação obtida pelo participante na primeira, segunda, terceira tentativa e média das tentativas foram: -0,225 correlação péssima, 0,431 correlação ruim, 0,412 correlação ruim e 0,336 correlação péssima, respectivamente.

### Influência da idade

A Tabela 2 apresenta a análise comparativa entre a pontuação média dos adultos menores que 22 anos e maiores que 22 anos. Não houve diferença com relevância estatística ao comparar os resultados.

**Tabela 2:** Análise comparativa entre a pontuação média dos adultos menores que 22 anos e maiores que 22 anos

Idade	Pontuação		Valor de p*
Até 22 anos (n=5)	Média	103,8	0,543
	Mediana	107,3	
	Desvio Padrão	16,3	
	Mínimo	84,0	
	Máximo	121,3	
Mais de 22 anos (n=4)	Média	110,5	
	Mediana	114,0	
	Desvio Padrão	14,8	
	Mínimo	90,7	
	Máximo	123,3	

* Teste T pareado

A Tabela 3 apresenta a análise comparativa entre as pontuações das crianças com tensão de lábios normal e adultos. Houve diferença com relevância estatística ao comparar os resultados das crianças e adultos em relação à primeira tentativa e à média das tentativas das crianças, tendo as crianças obtido menor pontuação do que os adultos.

**Tabela 3:** Análise comparativa entre as pontuações das crianças com tensão de lábios normal e dos adultos

Tentativa		Adulto (n=9)	Criança (n=4)	Valor de p*
1ª	Média	105,6	74,3	0,020
	Mediana	105,0	82,0	
	Desvio Padrão	17,7	13,3	
	Mínimo	69,0	59,0	
	Máximo	129,0	82,0	
2ª	Média	105,6	74,0	0,055
	Mediana	105,0	80,0	
	Desvio Padrão	22,5	28,9	
	Mínimo	73,0	34,0	
	Máximo	129,0	102,0	
3ª	Média	109,2	80,7	0,084
	Mediana	119,0	70,0	
	Desvio Padrão	23,2	18,5	
	Mínimo	70,0	70,0	
	Máximo	129,0	102,0	
Média das tentativas	Média	106,8	75,4	0,004
	Mediana	107,7	78,8	
	Desvio Padrão	15,1	12,1	
	Mínimo	84,0	58,0	
	Máximo	123,3	86,0	

* Teste T

### Influência da tensão labial

A Tabela 4 apresenta a análise comparativa entre a pontuação obtida pelas crianças com tensão de lábios normal e pelas crianças com tensão diminuída. As comparações não indicaram diferença com relevância estatística.

**Tabela 4:** Análise comparativa entre as pontuações obtidas por crianças com tensão normal e por crianças com tensão diminuída

Tensão labial	Características	1ª tentativa	2ª tentativa	3ª tentativa	Média
Normal	Média	74,3	74,0	80,7	75,4
(n=4)					
	Mediana	82,0	80,0	70,0	78,8
	Desvio Padrão	13,3	28,9	18,5	12,1
	Mínimo	59,0	34,0	70,0	58,0
	Máximo	82,0	102,0	102,0	86,0
Diminuída (n=5)	Média	76,3	63,3	94,0	75,4
	Mediana	85,5	54,5	98,0	78,0
	Desvio Padrão	34,3	34,3	34,7	16,1
	Mínimo	26,0	25,0	51,0	55,0
	Máximo	107,0	119,0	129,0	98,7
Valor de p*		0,927	0,623	0,578	0,995

* Teste T

### Influência do número de tentativas

A Tabela 5 apresenta a análise comparativa entre as tentativas realizadas pelas crianças e adultos. Apesar de apresentarem maior pontuação na terceira tentativa, não houve diferença significativa nas pontuações obtidas pelas crianças ou pelos adultos entre diferentes tentativas.

**Tabela 5:** Análise comparativa entre as pontuações obtidas no jogo nas diferentes tentativas realizadas pelas crianças e adultos

Adultos	Tentativa	Média	Desvio padrão	Valor de p*
1ª	105,6	17,7	1,000
2ª	105,6	22,5	
1ª	105,6	17,7	0,751
3ª	109,2	23,2	
2ª	105,6	22,5	0,558
3ª	109,2	23,2	
Crianças independente da tensão labial (n=9)	1ª	64,5	34,0	0,412
	2ª	79,0	39,3	
	1ª	64,5	34,0	0,420
	3ª	91,0	36,6	
	2ª	79,0	39,2	0,500
	3ª	91,0	36,6	
Crianças com tensão de lábios normal (n=4)	1ª	74,3	13,3	0,670
	2ª	74,0	28,9	
	1ª	74,3	13,3	0,673
	3ª	80,7	18,5	
	2ª	74,0	28,9	0,740
	3ª	80,7	18,5	
Crianças com tensão de lábios diminuída (n=5)	1ª	76,3	34,3	0,532
	2ª	63,3	34,3	
	1ª	76,3	34,3	0,557
	3ª	94,0	34,7	
	2ª	63,3	34,3	0,392
	3ª	94,0	34,7	

* Teste T-pareado

## DISCUSSÃO

### Análise da usabilidade

Nesta pesquisa foi possível obter dados preliminares sobre a usabilidade do dispositivo para reabilitação da força e mobilidade dos lábios associado a jogos digitais. Esse instrumento pode elevar a terapia fonoaudiológica a um nível mais lúdico, com potencial para promover maior adesão e motivação, principalmente do público infantil. Além disso, é possível que o terapeuta acompanhe e registre a evolução do paciente ao longo das terapias.

Os resultados do questionário SUS apontam que as respostas dos adultos sobre a usabilidade do dispositivo foram satisfatórias, considerando-se o ponto de corte de 68 pontos para este questionário^(26,27)^. Contudo, houve discordâncias nas questões *“preciso de ajuda para operar o instrumento”* e “*é preciso aprender muitas informações antes de usar o instrumento”*, indicando que o dispositivo pode não ser suficientemente intuitivo e que são necessárias alterações para facilitação da sua utilização. Dessa forma, pode-se considerar que existe a necessidade de melhorias no *design* e na interface do dispositivo para facilitar o uso, especialmente considerando o público infantil, que pode ter mais dificuldades em operá-lo de forma autônoma.

A média do escore total do teste indicou que o instrumento, na opinião dos participantes, apresentou boa usabilidade, e que o julgamento de usabilidade não está relacionado ao desempenho no jogo, haja vista a ausência de correlação entre o escore do SUS e a pontuação no jogo. É válido considerar que, embora validado e amplamente utilizado, o SUS apresenta limitações quando aplicado a dispositivos específicos de reabilitação, onde aspectos ergonômicos e funcionais podem ter um peso significativo na percepção de usabilidade; e que o ponto de corte original do instrumento pode não ser o mais adequado para este tipo de dispositivo. Não foram encontrados estudos que utilizaram o SUS na avaliação de instrumentos específicos de reabilitação labial, mas, no contexto da reabilitação fonoaudiológica, o escore obtido para um aplicativo destinado ao treino de deglutição, na percepção de usuários idosos, apresentou média de 56,0 e 70,8 pontos, respectivamente para idosos com menor e maior nível educacional e familiaridade no uso de aplicativos móveis^(28)^. Um outro aplicativo, destinado ao treino de fala, recebeu escore médio de 89,2, por voluntários saudáveis, sem alterações na fala^(29)^. A falta de estudos com equipamentos semelhantes ao desta pesquisa dificulta as comparações entre escores do SUS no contexto específico de reabilitação labial. No entanto, o escore obtido na presente pesquisa, 91,1, é bem mais elevado do que o ponto de corte, podendo ser considerado representativo de boa usabilidade.

Apesar de ser um passo relevante, a amostra de apenas 11 adultos na avaliação da usabilidade, pode ser considerada uma limitação para generalizações sobre a usabilidade do dispositivo. Além disso, a faixa etária da amostra que participou da avaliação da usabilidade, 20 a 41 anos, impede que os dados sejam generalizados para outras idades e a divisão dessa amostra entre gêneros e faixas etárias menores foi inviável diante do seu tamanho reduzido, sendo indicada a realização de futuras pesquisas que possam analisar com maior profundidade as percepções de usabilidade entre usuários.

Ressalta-se que a influência da familiaridade com jogos digitais não foi investigada nesta pesquisa. Essa familiaridade pode ter influenciado a percepção de usabilidade, uma vez que o jogo pode ser mais intuitivo para alguns perfis de usuário. Nesse sentido, o treinamento realizado com todos os participantes pode ter minimizado os efeitos da experiência prévia com jogos digitais. Mesmo não havendo relação com significância estatística entre o desempenho no jogo e a percepção de usabilidade indicada pelo participante, sugere-se que o tempo despendido semanalmente pelos participantes em jogos eletrônicos seja investigado nas próximas pesquisas.

### Influência da idade

Não houve diferença na pontuação entre adultos com menos ou com mais de 22 anos. O ponto de corte de 22 anos foi estabelecido por ser a mediana de idade da amostra de adultos. A literatura indica que diferenças de desempenho em jogos computacionais acionados pela língua podem existir quando comparadas faixas etárias mais distantes, como no estudo de Kothari et al.^(30)^, em os participantes com idades entre 21 e 35 anos apresentaram melhor desempenho do que aqueles na faixa entre 52 a 72 anos. Essa diferença pode ser justificada pela perda de massa muscular^(31)^, redução da velocidade e destreza^(32,33)^ típicas do envelhecimento. Não foram encontrados estudos semelhantes relacionados aos lábios, mas pesquisas apontam redução de força^(34)^ e de resistência^(34-36)^ labiais a partir dos 60 anos.

Na comparação entre adultos e crianças com tensão normal, a pontuação média das crianças foi menor. Esse achado pode ser explicado pelo desenvolvimento motor, tanto da morfologia muscular perioral, quanto do sistema nervoso central, caracterizado pelo aumento de diâmetro e mielinização dos axônios durante a infância e adolescência^(37)^. Um estudo realizado por Saitoh et al.^(38)^ associa a força labial às alterações hormonais que acompanham a maturação sexual e o crescimento somático da capacidade funcional, portanto a imaturidade estrutural das crianças deve ser considerada para explicar a diferença no desempenho. A maturação do sistema nervoso central também abarca o desenvolvimento de habilidades cognitivas e visomotoras relacionadas à realização de predições sobre a ocorrência e a duração de eventos futuros e programação do tempo de reação^(39)^, habilidades estas requeridas no jogo.

Verificou-se, ainda, que o tamanho do instrumento é uma variável a ser considerada na análise da força aplicada^(38)^. Portanto, o volume do instrumento utilizado na presente pesquisa pode ter influenciado na pontuação das crianças, que apresentam lábios com dimensões menores do que os dos adultos. Dessa forma, ressalta-se a necessidade de adaptação das dimensões do instrumento para o público infantil.

A influência do gênero do participante nos resultados não foi investigada nessa pesquisa, especialmente devido à amostra reduzida para ser subdividida por gênero. Contudo, pesquisas realizadas com um instrumento para reabilitação da língua por meio de jogos digitais não encontraram diferença entre os sexos para adultos^(12)^ ou crianças^(10)^. A literatura atribui melhor desempenho em jogos digitais para o sexo masculino, justificado pelo maior tempo geralmente despendido nessa atividade^(30)^, mas o instrumento utilizado nessa pesquisa tem um funcionamento atípico para todos os participantes, não dependente da destreza manual, o que minimiza a influência da familiaridade.

### Influência da tensão labial

Os testes realizados com as crianças indicaram que o instrumento é promissor e pode ser usado para essa população. Entretanto, visto o número reduzido de indivíduos nas amostras, a análise dos resultados deve ser interpretada com cautela, principalmente quando separados de acordo com a alteração da tensão labial. Considerando-se a média da pontuação, tanto as crianças quanto os adultos apresentaram pontuações semelhantes quando comparadas a primeira com a segunda tentativa, com pequeno aumento da pontuação na terceira tentativa. Contudo, os resultados observados em crianças com alteração da tensão labial não seguiram este padrão. Estas crianças tiveram piora do desempenho na segunda tentativa seguida de melhora na terceira. Maiores investigações precisam ser realizadas no intuito de compreender a interação entre os efeitos da fadiga e do aprendizado motor para esta população. A força solicitada no jogo era calculada individualmente no momento da calibração, considerando a força máxima de cada participante, o que pode explicar a ausência de diferença entre adultos de diferentes faixas etárias, bem como a ausência de diferença entre crianças com tensão normal e diminuída, visto que para aquelas com tensão diminuída o jogo exigia menor nível de força.

### Influência do número de tentativas

Apesar da ausência de diferença significativa entre as pontuações obtidas em cada tentativa, foi possível observar maior pontuação na terceira tentativa nos resultados dos adultos e das crianças no jogo, o que corrobora outros estudos^(12,40,41)^ com jogos computacionais associados a dispositivos acionados pela musculatura orofacial. Tais estudos^(12,40,41)^ associaram o aumento da pontuação nas sucessivas tentativas ao efeito de aprendizagem. De acordo com Huo e Ghovanloo (2010), para este tipo de atividade, os indivíduos ganham experiência rapidamente e melhoram o seu desempenho mesmo com um pequeno número de tentativas^(40)^. Uma vez que, nesta pesquisa, apenas três tentativas foram realizadas, sugere-se que futuras pesquisas explorem mais profundamente como esse efeito de aprendizagem ocorre ao longo de várias tentativas e se há um ponto de estabilização do desempenho.

É interessante mencionar que o treinamento realizado previamente à primeira tentativa foi importante para minimizar uma possível falta de compreensão dos participantes sobre como agir na dinâmica do jogo ou sobre como realizar o movimento com os lábios, o que poderia ter exacerbado o efeito do treinamento se o primeiro contato com o jogo tivesse ocorrido já na primeira tentativa.

Ao contrário do que foi verificado, esperava-se que a atenção e a motivação das crianças pudessem diminuir ao longo das três tentativas, pelo fato de o jogo ser idêntico em todas as tentativas, e que a fadiga muscular pudesse influenciar de forma negativa o desempenho dos participantes, especialmente as crianças com tensão muscular diminuída. No entanto, essas condições, se ocorreram, não foram suficientes para piorar o desempenho dos participantes, visto que a terceira tentativa apresentou o melhor desempenho em todos os grupos pesquisados.

### Limitações e sugestões para futuras pesquisas

O estudo é inovador e oferece *insights* promissores sobre o uso de jogos digitais para reabilitação orofacial. Uma limitação do estudo está relacionada ao tamanho da amostra, principalmente no que diz respeito às crianças com alteração de tensão labial. Outras limitações importantes referem-se à falta de medidas quantitativas de força, tempo de sustentação da contração e fadiga muscular. Tais medidas seriam importantes para uma avaliação mais detalhada da eficácia do dispositivo e para comparação com outros métodos de terapia. Dessa forma, a ausência dos dados quantitativos limita a compreensão dos benefícios fisiológicos do dispositivo em termos de reabilitação muscular, e indica a necessidade de novas pesquisas para suprir essa lacuna. A necessidade de adaptação para diferentes grupos populacionais também constitui fator que reduz a validade e a aplicabilidade imediata dos resultados. Outra limitação da pesquisa refere-se à pontuação diferenciada para alvos com variados níveis de dificuldade. Este constituiu um artifício implementado para aumentar a recompensa diante de alvos que requerem maior esforço, mas que dificultou a análise comparativa de desempenho entre os usuários. Nesse sentido, relatórios mais detalhados, incluindo informações sobre a quantidade de alvos de cada nível de dificuldade alcançados no decorrer do jogo devem ser incorporados no processamento do *software* para um *feedback* mais robusto do desempenho de cada usuário.

Por fim, pode-se citar também como limitação a ausência de informações sobre a familiaridade dos participantes com jogos digitais, cabendo ressaltar que o instrumento utilizado possui características próprias e um funcionamento incomum, não dependente da destreza manual, o que minimiza a influência da familiaridade com os *joysticks* dos *videogames* tradicionais.

Sugere-se que, em futuras pesquisas, o método seja utilizado em indivíduos com diferentes graus de alteração no tônus/tensão labial, em diferentes faixas etárias e com condições clínicas diversas, como paralisia facial e síndrome de Down, por exemplo, com alvos em diferentes patamares de força. Sugere-se, também, a incorporação de medidas quantitativas de avaliação de força e fadiga muscular. Além disso, sugere-se que sejam realizados estudos longitudinais, comparando o presente instrumento com métodos tradicionais da terapia fonoaudiológica, bem como a utilização de métodos objetivos para medir o entretenimento e interesse das crianças.

## CONCLUSÃO

O instrumento avaliado apresentou boa usabilidade na opinião dos adultos que testaram o equipamento. A correlação entre a pontuação dos adultos no jogo e a opinião sobre a usabilidade foi péssima ou ruim, evidenciando a falta de relação entre dificuldade em jogar e sucesso na execução das tarefas. Não houve diferença com relevância estatística na pontuação do jogo entre indivíduos adultos com mais e com menos de 22 anos. Na comparação entre crianças e adultos, as crianças obtiveram menor pontuação do que os adultos na primeira tentativa, bem como na média das tentativas. Não houve diferença com relevância estatística ao comparar a pontuação das crianças com tensão labial normal e tensão labial alterada. Não houve diferença significativa entre as pontuações obtidas pelas crianças ou pelos adultos nas diferentes tentativas.
